# Neutral ceramidase is a marker for cognitive performance in rats and monkeys

**DOI:** 10.1007/s43440-020-00159-2

**Published:** 2020-09-16

**Authors:** Liubov S. Kalinichenko, An-Li Wang, Christiane Mühle, Laila Abdel-Hafiz, Erich Gulbins, Johannes Kornhuber, André W. C. Oliveira, Marilia Barros, Joseph P. Huston, Christian P. Müller

**Affiliations:** 1 Department of Psychiatry and Psychotherapy, University Clinic, Friedrich-Alexander-University of Erlangen-Nuremberg, Schwabachanlage 6, 91054 Erlangen, Germany; 2 Center for Behavioral Neuroscience, Institute of Experimental Psychology, University of Düsseldorf, Düsseldorf, 40225 Germany; 3 Department of Molecular Biology, University of Duisburg-Essen, Essen, 45147 Germany; 4 Department of Surgery, College of Medicine, University of Cincinnati, 231 Albert Sabin Way, Cincinnati, OH 45267-0558 USA; 5 Department of Pharmacy, School of Health Sciences, University of Brasilia, Brasilia, DF 70910-900 Brazil; 6 Institute of Biology, Primate Center, University of Brasilia, Brasilia, 70910-900 Brazil

**Keywords:** Neutral ceramidase, Memory, Ceramide, Ventral mesencephalon, Rats, Non-human primates

## Abstract

**Background:**

Ceramides are lipid molecules determining cell integrity and intercellular signaling, and thus, involved in the pathogenesis of several psychiatric and neurodegenerative disorders. However, little is known about the role of particular enzymes of the ceramide metabolism in the mechanisms of normal behavioral plasticity. Here, we studied the contribution of neutral ceramidase (NC), one of the main enzymes mediating ceramide degradation, in the mechanisms of learning and memory in rats and non-human primates.

**Methods:**

Naïve Wistar rats and black tufted-ear marmosets (*Callithrix penicillata*) were tested in several tests for short- and long-term memory and then divided into groups with various memory performance. The activities of NC and acid ceramidase (AC) were measured in these animals. Additionally, anxiety and depression-like behavior and brain levels of monoamines were assessed in the rats.

**Results:**

We observed a predictive role of NC activity in the blood serum for superior performance of long-term object memory tasks in both species. A brain area analysis suggested that high NC activity in the ventral mesencephalon (VM) predicts better short-term memory performance in rats. High NC activity in the VM was also associated with worse long-term object memory, which might be mediated by an enhanced depression-like state and a monoaminergic imbalance.

**Conclusions:**

Altogether, these data suggest a role for NC in short- and long-term memory of various mammalian species. Serum activity of NC may possess a predictive role in the assessing the performance of certain types of memory.

**Electronic supplementary material:**

The online version of this article (10.1007/s43440-020-00159-2) contains supplementary material, which is available to authorized users.

## Introduction

Sphingolipids are among the most common lipids of the brain determining the structure of cellular membranes [1]. Besides the maintenance of cellular membranes integrity, sphingolipids, and particularly ceramide, determine the localization and functioning of proteins within the ceramide-enriched platforms of the membrane [2–4]. Changes in the composition of these platforms directly affect the affinity, signaling and internalization of protein receptors [5, 6] resulting in the development of certain physiological and pathological conditions. The constant level of ceramide is mediated by the ceramide rheostat including several metabolic pathways. One of them, the sphingomyelinase pathway, is based on the enzymes mediating ceramide generation from sphingomyelin, such as acid and neutral sphingomyelinases, and enzymes catalyzing ceramide degradation to sphingosine, such as acid and neutral ceramidases (AC and NC) [2–4]. The alterations in the levels and activities of all members of ceramide metabolism are involved in the pathogenesis of psychiatric disorders, such as depression [7–10], addiction [9, 11–15], as well as neurodegenerative disorders, such as Alzheimer’s and Parkinson’s disorder [16–20], and other disorders associated with memory loss [21, 22].

Memory is an essential neuropsychological mechanism enabling organisms to alter their behavior in accordance to past experience. This cognitive process is critical for survival as it guides actions determining life performance. Memory impairments might affect the cognitive abilities of humans at all stages of life: early disorders affecting child development, normal weakening of memory function during ageing, and neurodegenerative disorders associated with dementia in elderly people [23]. Extensive studies of recent decades showed that the mechanisms of learning and memory involve a variety of protein signaling cascades [23–25]. However, lately, the involvement of members of ceramide rheostat in the mechanisms of normal behavioral plasticity started to emerge. It was observed that the ceramides with various chain lengths, their precursors sphingomyelins, and enzymes determining ceramide generation from sphingomyelins, such as acid and neutral sphingomyelinases, significantly contribute to the development of unconditioned memory in rodents, non-human primates and humans [26–29]. The mechanism of involvement of the ceramide rheostat in the mechanisms of memory might be based on the alterations in the functioning of monoaminergic and glutamatergic systems [29]. However, the role of the enzymes controlling the degradation of ceramide to sphingosine, particularly NC, remains elusive.

Here, we have analyzed the contribution of NC to the mechanisms of learning and memory. We found evidence for a predictive role of NC activity in the blood serum for superior performers in a long-term object memory task in rats and in non-human primates. A higher NC activity in the ventral mesencephalon (VM) of rats, but not in other brain structures, predicted better performance in a spatial working memory test. In contrast, rats with bad long-term learning abilities in the object recognition test were characterized by high NC activity in the VM, which might be mediated by an enhanced depression-like trait and altered monoaminergic balance in rats.

## Materials and methods

### Experiment I: animals

Thirty male Wistar rats (aged 8–9 weeks) were purchased from Janvier (Le Genest St. Isle, France). Rats were grouped in five per cage and housed in Makrolon cages (Type IV; 60 × 38 × 20 cm) with controlled temperature and humidity conditions. All animals had free access to water and food on a 12-h/12-h light–dark cycle (lights on at 7 pm). They were allowed to adapt to the environment over 3 weeks and, then, handled for 5 min/rat/day for three consecutive days prior to experimentation. All procedures were followed by the Animal Protection Law of Germany and the European Communities Council 2010 Directive (2010/63/EU) and approved by the German Animal Protection Law Authorities (LANUV), Regierung von Düsseldorf.

### Behavioral test in rats

#### Short-term memory in the Y-maze

The wooden black Y-maze apparatus consists of three identical arms set at an angle of 120° from each other (16-cm wide, 50-cm long, and with walls 32-cm high) and a triangle-shaped central platform. The apparatus was placed in a sound-attenuating room and illuminated by diffuse white light (4.7 lx). The animals were placed in the center and were allowed to explore the Y-maze for 8 min. Entries to different arms were manually recorded. Successful entry into an arm was considered when a rat entered an arm with all four paws. The percentage of spontaneous alternation used as an index of short-term spatial memory was calculated using the following formula: Spontaneous alternation behavior (%) = Number of spontaneous alternations/(total number of entries − 2) × 100. Between sessions, the apparatus was cleaned with 70% ethanol in order to avoid any odor cues, and allowed to dry [30].

#### Long-term memory in the novel object recognition test

The same cohort of animals (*n* = 30) was tested for long-term memory in a novel object recognition task (NOR). An open field (OF) was made of gray polyvinyl chloride with 60 × 60 × 40 cm sidewall dimensions. The apparatus was placed in a sound-attenuating room with two spatial visual cues on different walls. A camera was mounted above the OF. Tracking and semi-automated analysis software (EthoVision X^®^ 8, Noldus, Netherland) was used. Diffuse white light provided even illumination with the intensity of ~ 4.7 lx at the center and 4.1 lx in the corners. Before the experiment, each animal was exposed to an empty OF for 10 min in order to habituate the animals. One day after habituation, an animal was placed inside the OF with two identical objects in the corners. The animal was allowed to explore the objects for a 3-min familiarization session. After a 48-h delay, a test trial was performed with one object being replaced by a novel object with a different shape. Rats were allowed to freely explore the familiar and novel objects for 4 min [30].

#### Elevated plus maze (EPM)

A plus-shaped maze was fixed 50 cm from the floor and contained two open arms (50 × 10 cm), two closed arms with surrounding walls (50 × 10 × 40 cm) and a center area (10 × 10 cm). The illumination conditions (open arm: 3 lx, closed arm: 14 lx) followed that of the previous protocol. A recording system was installed 2 m above the apparatus for recording the video and post-analyzing via the EthoVision XT software [31]. Rats were placed into the center facing an open arm and allowed to explore freely for 5 min. As the key parameter for analysis, the time spent in the open arms was measured. The apparatus was cleaned with 70% ethanol prior to each trial to eliminate possible odour cues.

#### Novelty suppressed feeding (NSF)

An unfamiliar OF (55 × 55 × 45 cm) with even illuminations (corners and center: ~ 6 lx) was used. A round, transparent dish (*d* = 7 cm) was placed in the center of the OF containing on a black rubber mat covering the floor of the OF. The apparatus was cleaned with 70% ethanol prior to each trial to eliminate possible odor cues. A recording system was installed 2 m above the apparatus to record the videos and post-analyzing via the EthoVision XT software. Twenty-four hours before the NSF, rats were food-deprived. The dish containing 2–3 lab chow pieces was placed in the center of the unfamiliar OF before the start of one trial. Then, animals were placed in one of the corners of the apparatus and allowed to explore freely for a maximum of 15 min. As soon as they were observed to eat, or the 15-min time limit was reached, the trial was terminated. Lab chow was changed, without repeating, in each trial. The latencies to start eating were analyzed as key depression-like behavioural marker [2, 9].

After the behavioral testing, rats were anesthetized with carbon dioxide. Blood and brain samples were collected. The blood samples were collected in 1.3-ml EDTA vial and centrifuged at 3000 RPM and room temperature for 5 min. The supernatants were collected into clean 500-µl polypropylene Eppendorfs. All samples were stored at − 80 °C until further processing.

### Monoamine analysis

For an estimation of the brain tissue monoamine levels and NC activity assays, the brains of rats were harvested and brain areas were dissected bilaterally: ventral striatum (VS), dorsal striatum (DS), dorsal hippocampus (DH), ventral mesencephalon (VM), and hypothalamus (Hyp). Tissue of one hemisphere was used for monoamine analysis. The tissue was homogenized in 0.5-M perchloric acid, centrifuged, filtered, and stored at − 80 °C until further analysis. All samples were analyzed using HPLC with electrochemical detection to measure dopamine (DA), serotonin (5-HT) and norepinephrine (NA) levels. The column was an ET 125/2, Nucleosil 120-5, C-18 reversed-phase column (Macherey & Nagel). The mobile phase consisted of 75-mM NaH2PO4, 4-mM KCl, 20-µM EDTA, 1.5-mM sodium dodecylsulfate, 100-µl/L diethylamine, 12% alcohol and 12% acetonitrile adjusted to pH 6.0 using phosphoric acid (Carl Roth GmbH). The electrochemical detector (Intro) was set at 500 mV vs. an ISAAC reference electrode (Antec) at 30 °C. This setup allows simultaneous measurement of DA, 5-HT, and NA [32, 33].

### Experiment II: animals

For the study with non-human primates, adult black tufted-ear marmosets (*Callithrix penicillata*) were used and delivered results: 11 males and 11 females, 4.5–8.0 years old and weighing 295–500 g. The phase of the estrous cycle of the females was not established, yet none were pregnant or had infants recently prior to or after the experiment. All subjects were pair-housed in standard home-cages (2 × 1 x 2 m) of a same colony room at the Primate Center of the University of Brasilia/Brazil. The marmosets were either born at this site or transferred to this location from other facilities in Brazil at least 5 years prior to the study. The colony room consisted of a semi-covered outdoor facility, so all animals had free access to shaded and uncovered areas with natural light, temperature and humidity conditions. Each home-cage had a nest-box, ropes, wood perches, a pellet dispenser and a tray for fresh food. This fresh diet was provided daily at 07:00 h consisting of pieces of fruits and vegetables, boiled eggs, nuts, and live mealworms and/or cooked chicken breast. Unconsumed items were removed at 17:00 h. Water and chow were available ad libitum. Housing and maintenance conditions complied with the regulations of the Brazilian Institute of Environment and Renewable Natural Resources (IBAMA). Animal numbers and the procedures held were approved by the Animal Ethics Committee of the University of Brasilia (no. 075/2018). All procedures were carried out in accordance with the Brazilian regulations for the scientific use of laboratory animals (Lei Arouca 11.794/2008), as well as the CONCEA/Brazil and NIH/USA guidelines for the care and use of laboratory animals.

### Behavioral test in non-human primates

Long-term memory was tested in marmosets using a NOR test. A white rectangular OF (130 × 75 × 40 cm) was used, as previously described [34, 35]. It had three aluminum sides, whereas the fourth side was made of transparent glass. The top was also made of glass and the bottom of wire-mesh. A guillotine-type door located centrally on one of the longer aluminum sides served as entry/exit point. The apparatus was placed in a test room illuminated with diffuse white light. The marmosets were transported to and from this location in an aluminum transportation cage (35 × 20 × 23 cm) that attached directly to the OF. A camera mounted above the arena and another one placed in front of its glass side were connected to a computer located in an adjacent observation room for remote tracking.

The subjects were initially habituated to an empty OF for 10 min. One day after habituation, each marmoset was given access to the apparatus, which now had two identical objects placed in different corners. The animal was allowed to freely explore the objects for a 10-min familiarization trial before returning to its home-cage. After a 6-h interval, the subject was taken to the OF for a test trial. One object had been replaced by a novel item with a different shape and color. The marmoset was allowed to freely explore the familiar and novel objects for 10 min and then taken back to its home-cage. The familiar/novel objects and their locations were altered between subjects. They had no apparent significance and could not be displaced by the marmosets. The apparatus and objects were cleaned between trials with a 70% ethanol solution.

The time physically and visually exploring each object and the time spent in motion were manually recorded by the investigator on an analysis software (AnyMaze^®^, Stoelting, USA). A discrimination index was calculated for the test trial to establish object recognition memory: novel object exploration time—familiar object exploration time/exploration time of both objects. A single 1.5-mL blood sample was taken from each subject 1 week before the behavioral trial, following the procedure described previously [35, 36].

### Ceramidase activity analysis

The activity of the NC and AC enzyme which converts ceramide to sphingosine was determined in rat brain samples and in the serum of rats and marmosets using the NBD-C12-Cer fluorescent substrate (Invitrogen/Life Technologies) as described previously [37].

### Statistical analysis

All quantitative data were expressed as mean ± SEM. Data were analyzed using one-way or two-way ANOVAs. To determine single group differences, pre-planned comparisons were calculated using Fisher’s LSD tests with Bonferroni correction where appropriate or with *t* tests. Pearson correlations were calculated for the interaction analysis in a correlation matrix. Non-human primate data were analyzed using *t* tests. Results were considered statistically significant at *p* ≤ 0.05.

## Results

### No association of serum NC activity with short-term memory performance in rats

**Fig. 1:**
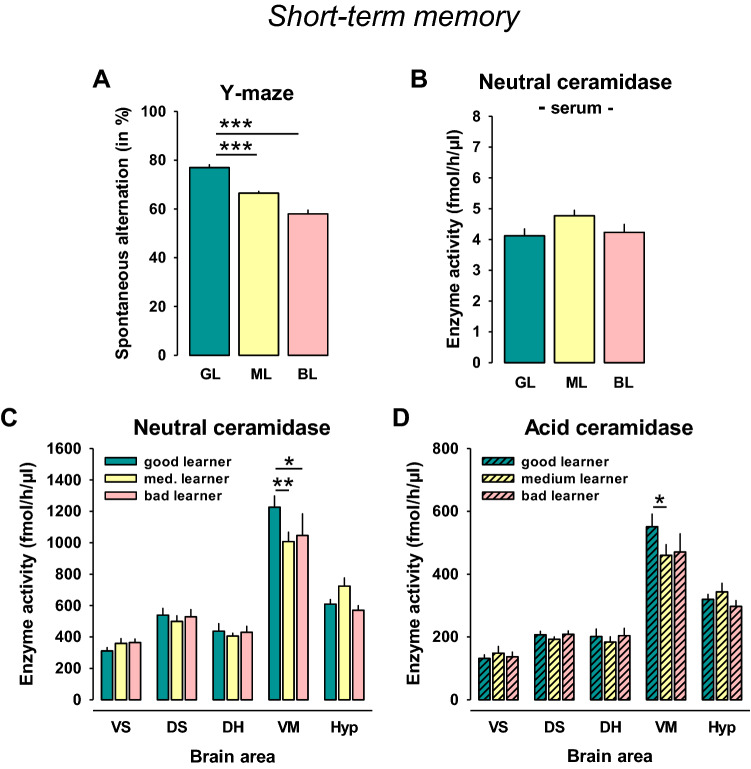
High neutral ceramidase (NC) and acid ceramidase (AC) activities in the ventral mesencephalon are associated with superior performance in a short-term memory task in rats. **a** Naïve rats were divided into 3 groups according to the percent of spontaneous alternations in a Y-maze test (*n* = 10/group). **b** No differences in serum NC activity between different learner types. **c** NC activity in the brain of different learner type rats. **d** AC activity in the brain of different learner type rats. Data are shown as mean ± SEM (*VS* ventral striatum, *DS* dorsal striatum, *DH* dorsal hippocampus, *VM* ventral mesencephalon, *Hyp* hypothalamus; *p < 0.05, **p < 0.01; ***p < 0.001)

Naïve rats were tested in the spontaneous alternation test and then divided into 3 groups according to the percent of spontaneous alternations (33:33:33%; good, medium, and bad learners; *n* = 10/group). Statistical analysis showed a significant difference between these groups (*F*_2,27_ = 63.189, *p* = 0.0001). The good learners performed better than medium learners (*p* = 0.0001) and bad learners (*p* = 0.0001; Fig. 1a). Serum NC activity did not differ between these groups (*p* > 0.05, Fig. 1b). AC activity was not detectable in the serum. In the Y-maze, there were high positive correlations between locomotor measures, such as distance moved, velocity, and arm entries (*p* < 0.05). All of which correlated negatively with spontaneous alternation rate and, thus, short-term memory performance (*r* = − 0.55; *r* = − 0.55, *r* = − 0.52; all: *p* < 0.05; Suppl. Tab. 1).

Analysis of brain NC activity showed no significant effect of the ‘learner type” (*F*_2,135_ = 0.595, *p* = 0.5532), but a significant difference between brain areas (*F*_4,135_ = 89.314, *p* = 0.0001), and no interaction effect (*F*_8,135_ = 1.704, *p* = 0.1028). The NC activity in the areas was VM ≫ Hyp > DS > DH > VS (Fig. 1c). Pre-planned comparison for single brain areas showed higher NC activity in the VM of good vs medium (*p* = 0.0096) or bad learners (*p* = 0.0398, Fig. 1c). However, no significant correlation between brain area and serum NC activity was found (*p* > 0.05, Suppl. Tab. 1).

Analysis of brain AC activity showed no significant effect of the “learner type” (*F*_2,135_ = 0.780, *p* = 0.4605), a significant difference between brain areas (*F*_4,135_ = 89.495, *p* = 0.0001), but no significant interaction effect (*F*_8,135_ = 1.033, *p* = 0.4141; Fig. 1d). The AC activity in the areas was VM ≫ Hyp > DS = DH > VS (Fig. 1d). Pre-planned comparison for single brain areas showed higher AC activity in the VM of good learners compared to medium learners (*p* = 0.0281) and a trend towards bad learners (*p* = 0.0589, Fig. 1d).

These results suggest that superior performance in a short-term memory task is associated with enhanced NC and AC activity in the VM, a brain region that showed the highest ceramidase activity of all investigated brain areas. No such relationship was found for serum NC activity.

### High serum NC activity is associated with superior long-term memory performance in rats

**Fig. 2:**
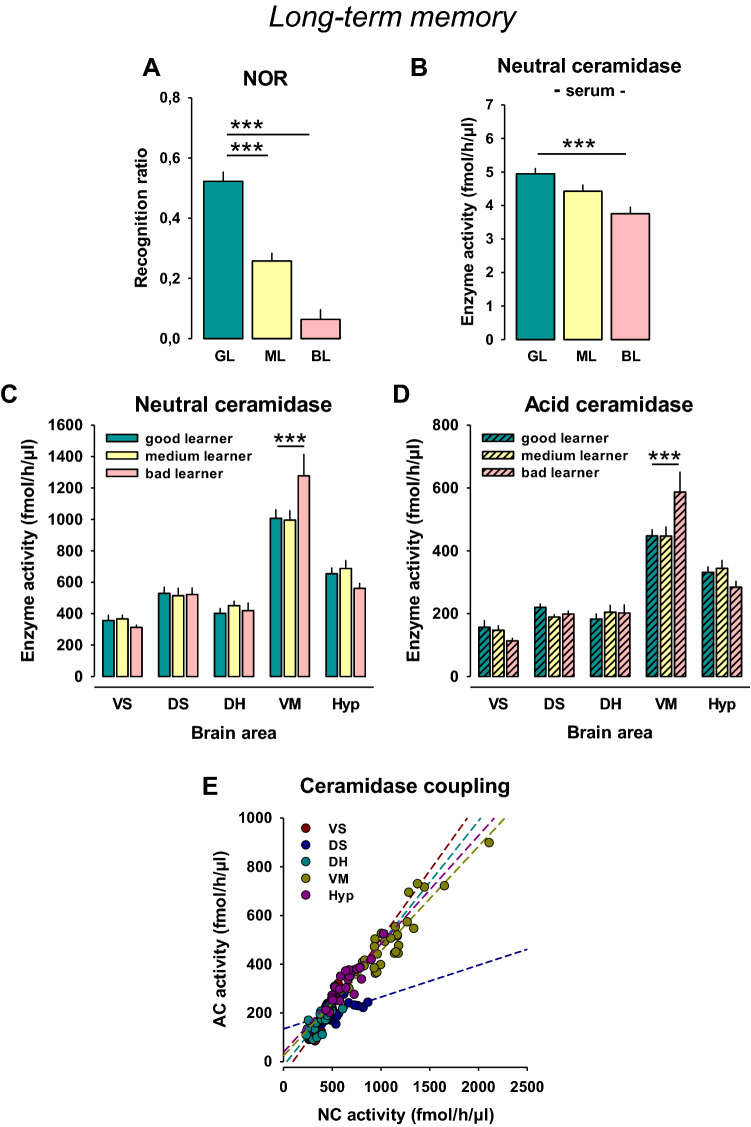
High serum neutral ceramidase (NC) activity is associated with superior performance in a long-term memory task in rats. **a** Naïve rats were divided into three groups according to the percent of spontaneous alternations in a novel object recognition test (*n* = 10/group). **b** Serum NC activity in different learner types. **c** NC activity in the brain of different learner type rats. **d** AC activity in the brain of different learner type rats. Data are shown as mean ± SEM (*VS* ventral striatum, *DS* dorsal striatum, *DH* dorsal hippocampus, *VM* ventral mesencephalon, *Hyp* hypothalamus; ***p < 0.001)

Rats were divided into 3 groups according to the recognition ratio which measured NOR performance (33:33:33%; good, medium, and bad learners; *n* = 10/group). Statistical analysis showed a significant difference between these groups (*F*_2,27_ = 59.122, *p* = 0.0001). The good learners performed better than medium learners (*p* = 0.0001) and bad learners (*p* = 0.0001; Fig. 2a). Serum NC activity differed significantly between these groups (*F*_2,27_ = 11.092, *p* = 0.0003), in that good learners had a higher activity than bad learners (*p* = 0.0001, Fig. 2b). In the NOR test, there was a positive correlation between early exploration time for objects and later discrimination rate (*r* = 0.5; *p* < 0.05). Y-maze short-term memory did not correlate with NOR long-term memory performance (*r* = − 0.05, *p* > 0.05). Interestingly, a good short-term memory in the Y-maze may have been the reason for less object exploration in the NOR (*r* = − 0.5, *p* < 0.05), and, thus, a not optimal long-term memory retrieval. This may be one reason for a lack of relationship between short- and long-term memory performance in this study. However, there was a strong positive correlation between serum NC activity and superior long-term memory performance in the NOR (*r* = 0.56, *p* < 0.05; Suppl. Tab. 1).

Analysis of brain NC activity showed no significant effect of the ‘learner type” (*F*_2,135_ = 0.379, *p* = 0.6852), but a significant difference between brain areas (*F*_4,135_ = 93.996, *p* = 0.0001), and a significant interaction effect (*F*_8,135_ = 2.740, *p* = 0.0078; Fig. 2c). Pre-planned comparison for single brain areas showed lower NC activity in the VM of good vs. bad learners (*p* = 0.0008, Fig. 2c). A correlation analysis of serum and brain NC activity over all animals tested suggested a significant positive relationship between NC activity in the serum and VS (*r* = 0.357, *p* = 0.05), but no significant relationship to other brain areas (*p* > 0.05).

Analysis of brain AC activity showed no significant effect of the ‘learner type” (*F*_2,135_ = 0.281, *p* = 0.7553), but a significant difference between brain areas (*F*_4,135_ = 100.979, *p* = 0.0001), and a significant interaction effect (*F*_8,135_ = 3.481, *p* = 0.0011; Fig. 2d). Pre-planned comparison for single brain areas showed lower AC activity in the VM of good learners compared to bad learners (*p* = 0.0001; Fig. 2d).

These results suggest that superior performance in a long-term memory task is associated with enhanced serum NC activity. This association was not found at brain region level, where good learners showed a reduced NC and AC activity in the VM.

### Ceramidase coupling

A correlation analysis of brain NC and AC activity revealed significant correlations in the VS (*r* = 0.88, *p* < 0.05), DS (*r* = 0.54, *p* < 0.05), DH (*r* = 0.86, *p* < 0.05), VM (*r* = 0.92, *p* < 0.05), and Hyp (*r* = 0.89, *p* < 0.05) (Fig. 2e). These findings may suggest a strong coupling of both enzymes in the brain.

### Local NC activity and emotional status

**Fig. 3:**
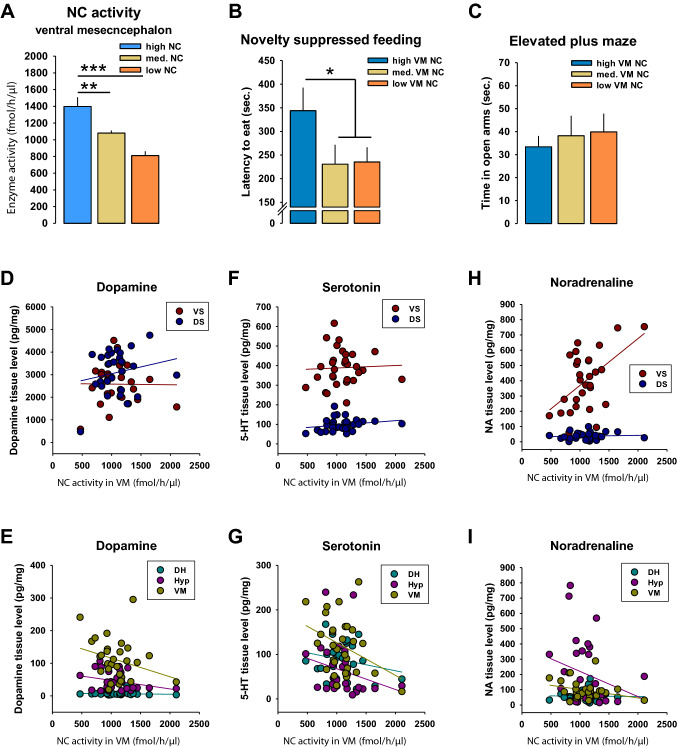
Ventral mesencephalon (VM) neutral ceramidase (NC) activity is associated with depression-like, but not anxiety-like behavior in rats. **a** Naïve rats were divided into 3 groups according to their NC activity in the VM (*n* = 10/group). **b** Enhanced depression-like behavior in animals with high VM NC activity. **c** No differences in anxiety in the elevated plus maze test. **d**–**i** Correlations between NC activity in the VM and tissue levels of dopamine, serotonin and noradrenaline. Data are shown as mean ± SEM (*VS* ventral striatum, *DS* dorsal striatum, *DH* dorsal hippocampus, *VM* ventral mesencephalon, *Hyp* hypothalamus; *p < 0.05, **p < 0.01; ***p < 0.001)

We found that bad long-term memory was associated with enhanced NC activity in the VM of the brain. We analyzed whether NC activity in the VM would predict emotional behavior and monoaminergic innervation of target brain regions. Animals were subdivided in high, medium and low VM NC activity (*n* = 10/group) and depressive behavior in the NSF test, as well as anxiety-associated behavior in the EPM test were compared. The group split yielded a significant difference between high vs. medium (*t* = 3.042, *p* = 0.0082) and high vs. low NC activity (*t* = 5.381, *p* = 0.0001) in the VM (Fig. 3a). We found that high NC animals showed a significantly higher latency to start feeding behavior in the NSF test than the medium and low NC animals combined (*t* = 2.285, *p* = 0.031; Fig. 3b). No difference was found between groups in open arm time of the EPM test (*p* > 0.05; Fig. 3c). There were no significant correlations between depression-related behaviors in the NSF and anxiety-related behaviors in the EPM (*p* > 0.05; Suppl. Tab. 1).

Altogether, these findings suggest that a bad long-term memory is associated with an enhanced depression-like state, but not with anxiety, which is possibly mediated by enhanced NC activity in the VM. Our results are in line with the previous data showing that the depression-like phenotype is associated with enhanced activities of NC and AC [10].

### NC activity and brain monoamines

The VM is a region, which contains the origin of numerous ascending modulatory neurotransmitter systems, which control arousal, attention, and emotional state [38, 39]. We tested whether NC activity in the VM would predict DA, 5-HT or NA levels in target areas of the respective projections. A correlational analysis did not find a relationship for DA (*p* > 0.05; Fig. 3d, e). It showed a significant inverse correlation of NC activity in the VM with local 5-HT levels (*r* = − 0.37, *p* < 0.05; Fig. 3), but a positive correlation with NA levels in the ventral striatum (VS) (*r* = 0.52, *p* < 0.05; Fig. 3h). These findings may suggest some coupling of NC activity in the VM with the control of local 5-HT and target area NA levels. A correlation analysis showed a high coupling of hypothalamic AC activity with NA (*r* = 0.38) and 5-HT (*r* = 0.49) levels in the VS, and with DA (*r* = 0.5) and 5-HT (*r* = 0.36) levels in the DS (*p* < 0.05). Interestingly, there was a negative correlation between hypothalamic AC activity and local levels of NA (*r* = − 0.53), DA (*r* = − 0.42) and 5-HT (*r* = − 0.46) (all: *p* < 0.05; Suppl. Tab. 1).

### High serum NC activity is associated with superior long-term memory performance in monkeys

**Fig. 4:**
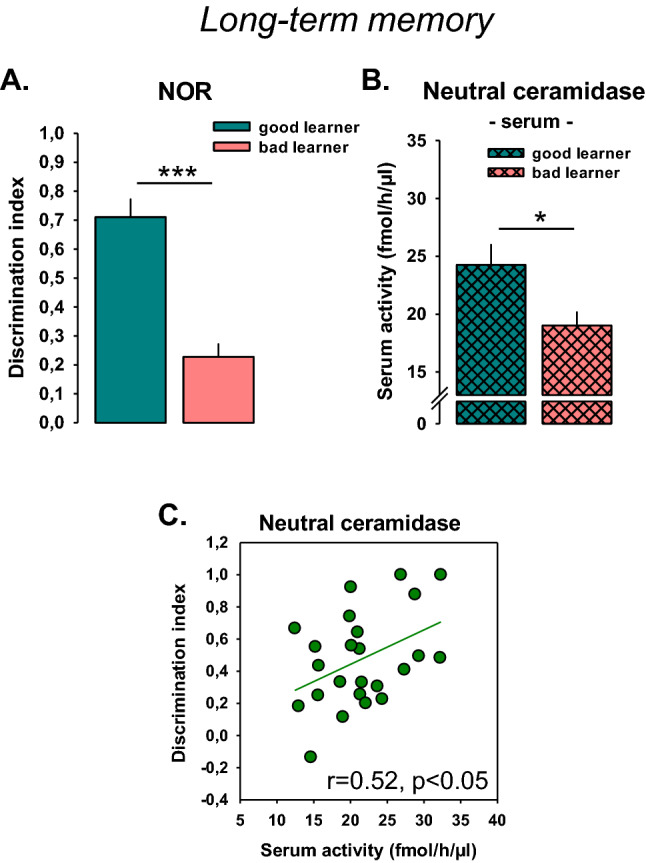
Blood serum activity of neutral ceramidase (NC) is a predictor for novel object recognition long-term memory performance in non-human primates (*Callithrix penicillata*). Data are shown as mean ± SEM. **a** The subjects (*n* = 22) were divided in two groups of good and bad learners by median split (50:50%). **b** Corresponding serum NC activity. **c** Animals with high serum NC activity showed a better long-term memory performance compared to animals with low NC (*p < 0.05, ***p < 0.001; *r* Pearson correlation)

Based on our findings in rodents, we hypothesized that superior performance in long-term memory would be associated with high serum NC activity in non-human primates. To test this, we analyzed the activity of NC the blood serum of *C. penicillata* and compared it to the performance in a NOR memory task. Male and female subjects (50:50, *n* = 22) were exposed to one object in an OF arena during a familiarization trial. After a delay of 6 h, they were exposed to the same object and a novel one. Memory of the first object was measured by object exploration and expressed as discrimination index. As the number of test subjects was limited in this study, only a median split in two groups of good vs. bad learners was performed. Good learners differed significantly from bad learners in the NOR performance (*t* = 6.3278, *p* = 0.0001; Fig. 4a). Good learners showed significantly higher serum NC activity than bad learners (*t* = 2.4961, *p* = 0.0214; Fig. 4b). Thereby, serum NC activity was a good predictor for NOR long-term memory performance as shown in a correlation analysis (*r* = 0.52, *p* < 0.05; Fig. 4c). Also in monkey serum, there was no detectable AC activity.

Altogether, these data confirm a positive relationship between serum NC activity and performance in a NOR long-term memory task also in non-human primates.

## Discussion

The ceramide rheostat is a crucial mechanism of cell membrane integrity determining synaptic appearance and signaling [40]. High abundance of ceramides, their precursors and metabolites in the brain [41] determines the specific involvement of these molecules in the pathogenesis of several psychiatric disorders [2, 7, 9–20]. Recent data show that the ceramide system also contributes to the mechanisms of memory performance both under physiological conditions [26–29] and in patients with neurodegenerative disorders associated with cognitive decline [17, 19, 21, 22]. Here, we showed the predictive role of NC, one of the crucial enzymes of the sphingomyelinase pathway of ceramide metabolism, for superior long-term memory performance of rats and non-human primates in novelty-based memory tests. Our data allow proposing that NC activity in the peripheral blood might be used as a potential biomarker for certain types of cognitive performance. In addition, high NC activity in the VM, but not in other brain structures or blood, was associated with good spatial short-term memory in naïve rats. On the contrary, low long-term object recognition memory performance in these rats was accompanied by high activity of NC in the VM, which might be explained by the high depression-like traits of these animals. Altogether, our data suggest a role of NC in learning and memory and show a possible predictive role of NC enzyme activity in blood serum for long-term memory performance.

NC is one of the crucial enzymes of ceramide metabolism expressed in mitochondria and the plasma membrane [42, 43] of various mammalian tissues, such as the brain, liver, kidney, pancreas, heart, and skeletal muscle of rodents and humans [44–47]. It may be proposed that the observed predictive role of NC in the peripheral blood of mammals for superior memory performance might be determined by its biological function. It is well known that the balance between programmed cell death and neurogenesis is a crucial mechanism of learning and memory in the adult brain [47]. Ceramide was shown to enhance programmed cell death via apoptosis [48–50]. In contrast to ceramide, its metabolite sphingosine-1-phosphate protects cells from apoptosis, and promote generation of new cells and their survival [51]. NC converts ceramide into less toxic metabolites [52] and, thus, might inhibit ceramide-induced apoptosis. Recent studies showed that cells overexpressing NC are protected against ceramide C6 and C2-induced apoptosis [44, 53, 54]. No literature data have shown a direct contribution of NC to neurogenesis yet. However, ceramides were shown to aggravate various stages of neurogenesis [2]. Thus, the reduction of ceramide levels mediated by NC might enhance neurogenesis. Altogether, high NC activity might increase the rate of newborn neurons in mammalian brain and, thus, enhance learning abilities and memory performance. This mechanism might explain the observed in our study superior long-term memory performance in the novelty-based tests in rats and non-human primates with higher activity of serum NC.

The analysis of the interaction between NC brain activity and memory performance in rats revealed that the activity of NC only in the VM, but not in other brain structures, significantly differs between good and bad learners. This specificity might be related to the role of the VM in the processes of memory and learning. The VM is an area containing the neurons of many monoaminergic systems, including dopaminergic, serotonergic and noradrenergic neurons. Thus, it is considered to be involved in the mechanisms of reinforcement learning and reward prediction [38, 39]. However, there is also an important contribution of the VM to the mechanisms of novelty recognition memory. The dopaminergic neurons project to several brain structures including hippocampus, which contributes to learning processes. It is proposed that the hippocampus and a part of VM, ventral tegmental area, act as a dynamical loop, particularly during the presentation of novel stimuli [55–58]. Presentation of a significant novel event leads to the activation of the ventral tegmental area resulting in the enhanced dopaminergic input to the hippocampus [59, 60]. DA was shown to be a key modulator for long-term potentiation the hippocampal CA1 region. Novel experience can activate dopaminergic neurons in the VM. Memory for novelty can be attenuated when hippocampal DA receptors are blocked [61]. Interestingly, similar processes are typical for the spatial novelty test [61], which confirms our data on the importance of NC specifically in the VM for the spontaneous alternation test. Altogether, these data support a crucial role of the VM for spatial novelty learning and memory. Even though the role of the VM during memory development is proposed to be mostly related by the pronounced dopaminergic input from this brain structure [58, 60], our data did not reveal significant correlations between tissue DA level and NC activity in this brain structure. Thus, it might be suggested that altered NC activity does not directly affect the tissue DA levels in target structures, but might influence DA release and synaptic throughput, which determines superior memory performance. However, it should be noted that correlational analysis over the whole population showed only a moderate and non-significant relationship. This may suggest that NC activity in the VM determines extreme good/bad performers rather than a continuous distribution.

Interestingly, superior performance of rats in the short-term vs. long-term NOR tests was associated with opposite changes in NC activity in the VM. Good learners in the test for short-term memory were characterized by high VM NC activity, which might be explained by the protective role of NC against ceramide-induced apoptosis as mentioned above. On the contrary, superior long-term NOR memory was accompanied by lower VM NC activity. This interaction might be associated with the enhanced depression-like trait in the rats with high NC activity in the VM. It is well known that depression might significantly impair long-term memory in humans and rodents [62–64]. Previous studies have shown the involvement of various ceramides, their precursors sphingomyelins, and enzymes mediating ceramide synthesis sphingolipid in depression [2, 8–10, 41, 65]. Thus, it may be suggested that the contribution of NC to the mechanism of depression-like behavior might affect long-term memory performance in rats. However, the mechanisms of an NC involvement in the pathogenesis of depression followed by memory impairments still have to be elucidated. It might be associated with the involvement of the ceramide system in the balance of apoptosis and neurogenesis at various stages. Low activity of NC in the brain might enhance neuronal survival [54, 66]. Previous studies revealed that mouse embryonic fibroblast cells with knocked out NC (NC^−/−^ cells) are protected from cell death induced by endoplasmatic reticulum stress after the administration of 2-deoxyglucose and antimycin A as compared to wild-type cells. Higher level of autophagy in these cells may promote cell survival during stress via degradation of misfolded proteins and up-regulation of autophagy flux [66]. Thus, lack of NC in the VM might possibly lead to higher neuronal survival, and, therefore, prevent the development of depression and enhance memory performance in the NOR test as shown in our study on rats.

It should be noted that our study on rats did not show significant correlations between the activity of NC in the serum and the VM as well as other brain structures except for the VS. Thus, even though the alterations in NC activity in the serum might predict the performance of rats in memory tests based on novelty, these changes do not reflect the changes in the brain. This might possibly be related to the presence of different forms of NC in the tissues and serum. It was proposed that the long form of NC is mostly located in the plasma membrane, while the short form is predominantly released [43]. However, additional studies of the possible differences and functions of these two forms of NC are required.

Thus, the present data suggest a contribution of the enzyme mediating ceramide degradation, NC, in the mechanisms of learning and memory in rats and non-human primates. High activity of NC in the blood serum predicted superior performance of long-term object memory in these species. Even though NC activity in the serum and VM of rats did not correlate, higher NC in the VM of rats predicted superior performance in a short-term spatial memory test for working memory. On the contrary, high NC activity in the VM of rats predicted worse performance of long-term object memory, which might be mediated by the enhanced depression-like trait of these animals. Altogether, these data suggest a new sphingolipid mechanism of learning and memory performance in various mammalian species mediated by NC. Serum activity of NC may possess a predictive role in the assessing the performance of certain types of memory.

## Electronic supplementary material

Below is the link to the electronic supplementary material.

Supplementary material 1 (PPTX 72 kb)
