# Supplementary material for: Neutral ceramidase is a marker for cognitive performance in rats and monkeys
Source: Pharmacol Rep. 2020 Sep 16;73(1):73–84. doi: 10.1007/s43440-020-00159-2 (PMC7862079; doi:10.1007/s43440-020-00159-2)

## Slide 1
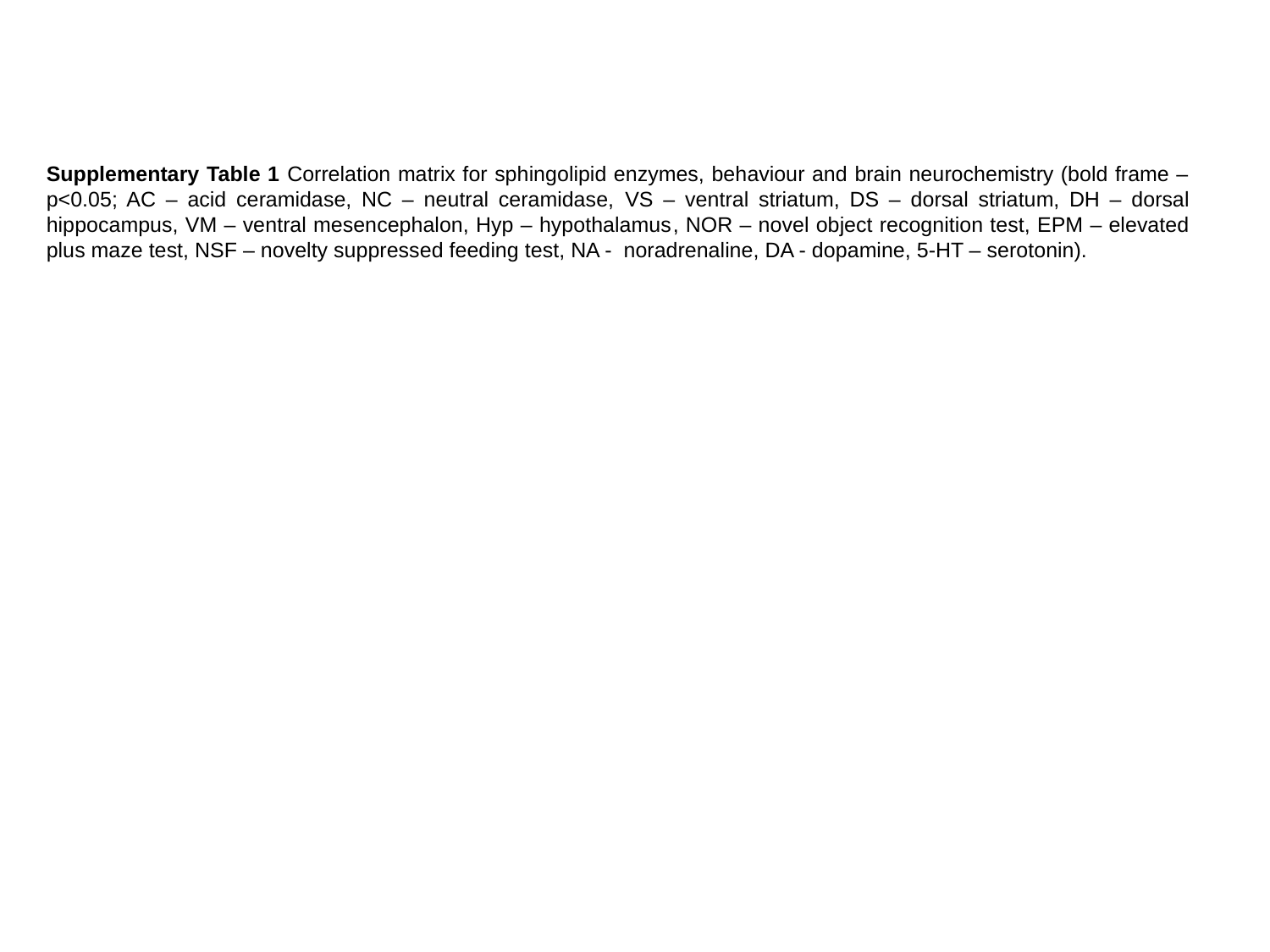

Supplementary Table 1 Correlation matrix for sphingolipid enzymes, behaviour and brain neurochemistry (bold frame – p<0.05; AC – acid ceramidase, NC – neutral ceramidase, VS – ventral striatum, DS – dorsal striatum, DH – dorsal hippocampus, VM – ventral mesencephalon, Hyp – hypothalamus, NOR – novel object recognition test, EPM – elevated plus maze test, NSF – novelty suppressed feeding test, NA - noradrenaline, DA - dopamine, 5-HT – serotonin).

## Slide 2
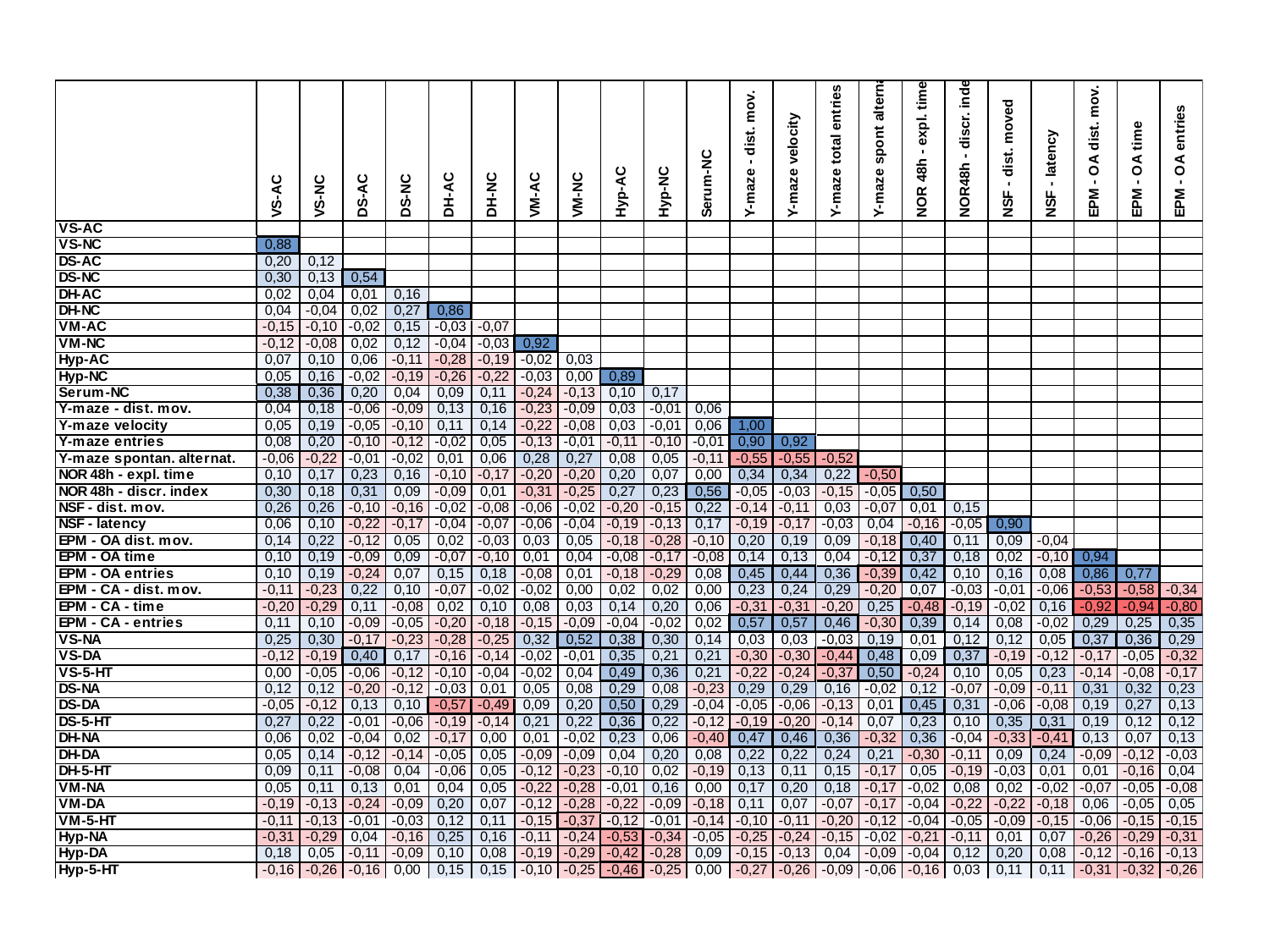

## Slide 3
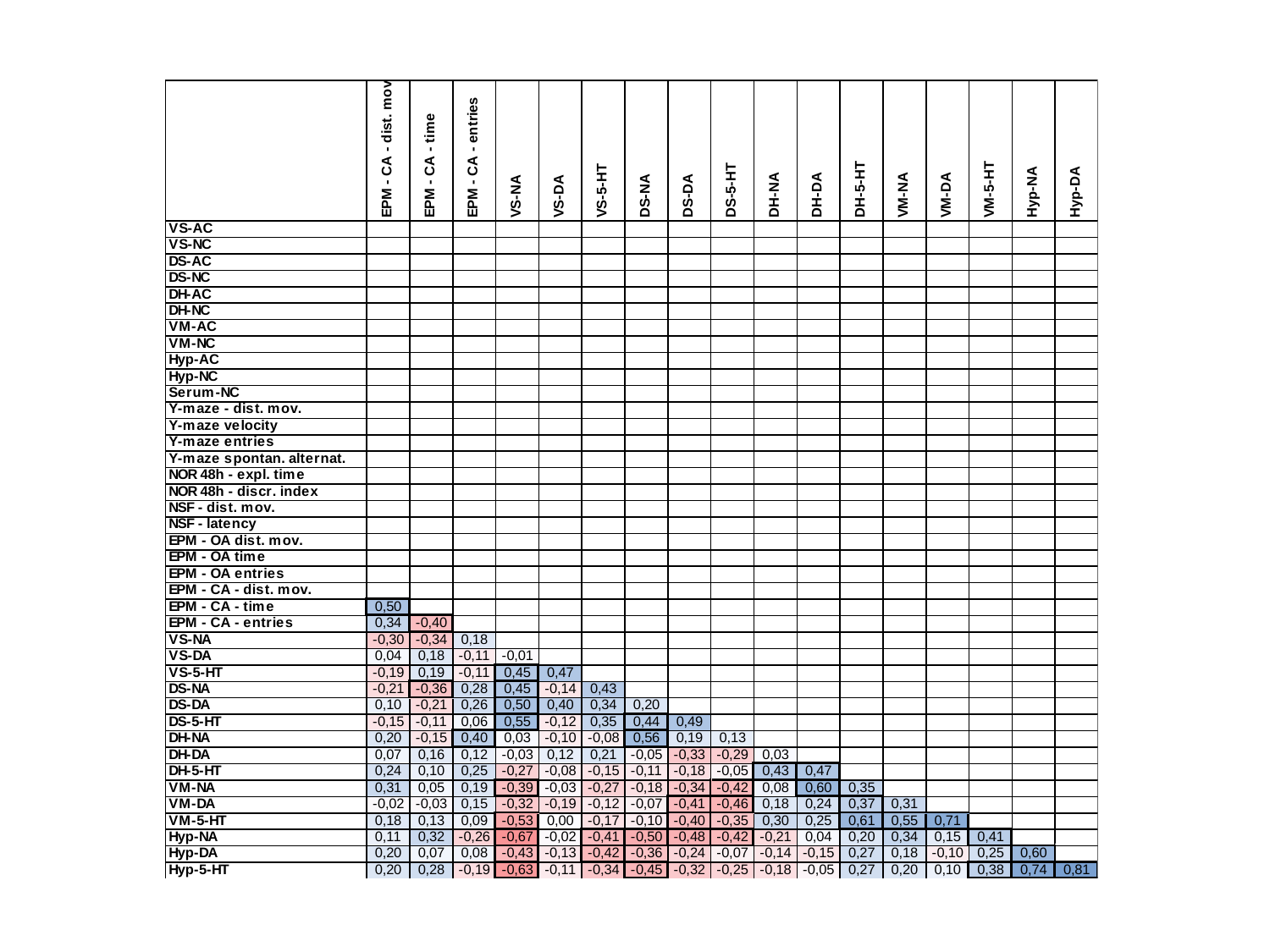

Supplement: Supplementary file 1 — Supplementary material 1 (PPTX 72 kb) [file 43440_2020_159_MOESM1_ESM.pptx]
